# Photocatalytic degradation of methylene blue using a Cu^2+^-modified bimetallic titanium-based metal organic framework (MIL-125) photocatalyst with enhanced visible light activity

**DOI:** 10.55730/1300-0527.3695

**Published:** 2024-04-30

**Authors:** Gülsüm ÖZÇELİK, Ferda CİVAN ÇAVUŞOĞLU, Şahika Sena BAYAZİT, Şeyma ÖZKARA AYDINOĞLU

**Affiliations:** 1 Department of Chemical Engineering, Faculty of Engineering and Architecture, İstanbul Beykent University, İstanbul, Turkiye; 2 Institute of Nanotechnology and Biotechnology, İstanbul University-Cerrahpaşa, İstanbul, Turkiye

**Keywords:** MIL-125, photodegradation, visible light induced, metal organic framework, titanium

## Abstract

Cu-modified TiO_2_ nanoparticles derived from MIL-125 were prepared by solvothermal method for the photocatalytic degradation of methylene blue under visible light illumination. For boosting the photocatalytic performance as well as the physicochemical properties of bare sample, 2 wt % Cu^2+^ ions were integrated into the nodes of the MIL-125 framework. The results showed that incorporation of 2 wt % Cu^2+^ ions into the MOF framework had significant effects on the crystallographic structure and morphological and optical properties of photocatalytic samples, as well as catalytic activity for the methylene blue degradation reaction. The high activity profile of Cu-modified TiO_2_ nanoparticles derived from MIL-125 might be attributed to the increased thermal stability, lower band gap energy, and smaller crystallite size of the sample. Activity tests were carried out at five varying MB initial concentrations and four different pH values. According to the findings, an increase in initial dye concentration resulted in a decrease in degradation efficiency. It was observed that increasing the pH value in the range of 3–11 initially led to higher degradation rates until pH 7, after which the degradation rate began to decline.

## 1. Introduction

Water pollution, causing considerable damage to human health and the environment, is a critical problem faced by the world at present. Synthetic dyes, which are hazardous, mutagenic, and generally nonbiodegradable, are severe contributors to water pollution as the wastewater effluents produced by the textile industry contain vast amounts of these dyes. Hence, eliminating pollutants from water is an urgent and important issue for human sustainability [1–3].

Several techniques such as precipitation, membrane filtration, coagulation, and adsorption have been used to eliminate dye contaminants from wastewater [4, 5]. However, these methods have been ineffective as the dye is not degraded; its concentration in water is only decreased by transforming it from one state to another, inadvertently generating new forms of pollution [6].

Lately, advanced oxidation processes for the elimination of pollutants from wastewater have been the center of attention as these processes are environmentally friendly and cost-effective and can reduce various dyes or organic pollutants existing in wastewater [6]. Photocatalytic degradation of dyes has arisen as an economical and environmentally sustainable alternative amongst several advanced oxidation processes as it uses renewable, unlimited solar energy in breaking down harmful dyes in wastewater, transforming them into harmless chemicals [7–10].

Metal-organic frameworks (MOFs) are a category of porous substances, featuring crystalline frameworks in three dimensions made up of metal units and organic ligands [11,12]. Recently, they have attracted widespread attention owing to their notable characteristics such as adjustable pore dimensions, precisely defined pore structures, and extensive surface areas [13–15]. Among the several sorts of MOFs, titanium-based metal-organic frameworks (Ti-MOFs) stand out as particularly appealing, primarily due to their favorable redox capabilities and robust metal-ligand bonding, contributing to a rigid framework [16–18].

MIL-125 is a Ti-MOF originating from the combination of titanium ions and organic ligands with carboxylate functional groups [19]. There are several studies in the literature reporting degradation of various dyes by MIL-125 MOFs [20–24]. Hu et al. [20] stated that high surface area MIL-125(Ti)-modified ZnIn_2_S_4_ photocatalysts displayed superior photocatalytic activity for the photocatalytic degradation of rhodamine B dye under visible-light illumination. Abdelhameed et al. [21] investigated the visible light photocatalytic behavior of nanocomposite NH_2_-MIL-125 samples encircled with Ag_3_PO_4_ nanoparticles in the degradation of rhodamine B and methylene blue, and found that Ag_3_PO_4_ nanoparticles lowered the optical band gap of the NH_2_-MIL-125. Emam et al. [22] studied MIL-125-NH_2_ nanoparticles doped with Ag_2_WO_4_ and Ag_3_VO_4_. These doped nanoparticles exhibited enhanced degradation capabilities for rhodamine B and methylene blue dyes when subjected to both UV and visible light. The presence of the nanoparticles led to a notable improvement in the degradation of both dye compounds. Zhai et al. [23] investigated the photocatalytic degradation of rhodamine B over Ag_3_VO_4_/MIL-125(Ti) photocatalysts under visible light irradiation. They demonstrated that the photocatalytic efficiency of composites was much higher than that of pure Ag_3_VO_4_ and MIL-125(Ti). Yuan et al. [24] prepared MIL-125 doped with reduced graphene oxide and Ag nanoparticles and tested the activity of the samples in the photocatalytic degradation of rhodamine B dye. They reported that the photodegradation rate of rhodamine B on MIL-125 modified with graphene and Ag was 1.62 times greater than that of bare MIL-125.

MOF crystals decompose at high temperatures and lose their crystallinity and transform into worthless materials. The controlled pyrolysis of MOFs can provide new and multifunctional materials [25]. MOF-derived oxides of transition metals are among these materials. They have numerous advantages, such as adjustable porosity and reduced electron transmission distance [26]. They can be used as very effective photocatalysts for water treatment.

Based on a comprehensive analysis of the literature, the current study focused on the design and development of Cu-modified TiO_2_ nanoparticles derived from MIL-125 prepared by solvothermal method. Aiming to enhance the photocatalytic performance of TiO_2_ nanoparticles derived from MIL-125, 2 wt % of Cu^2+^ ions were incorporated into the nodes of MOF frameworks to allow fine-tuning of the physicochemical properties of photocatalysts. The synthesized particles were tested by methylene blue dye degradation reaction under visible light. To determine the effect of Cu^2+^ ion incorporation into the MOF framework on Ti crystalline phases, X-ray diffraction (XRD) analyses were performed. The structural properties of MOF crystals were analyzed by FTIR. UV–vis diffuse reflectance spectra (UV–vis DRS) tests were conducted in order to characterize the optical absorption properties of photocatalyst samples. Thermal gravimetric analysis (TGA) was performed to explore the thermal behavior of MIL-125 samples. Scanning electron microscopy (SEM) and energy dispersive X-ray (EDX) analyses were conducted to gather insights into the distribution of metals over the catalyst surface.

## 2. Experimental

### 2.1. Materials

Titanium(IV) isopropoxide (C_12_H_28_O_4_Ti, Sigma-Aldrich), terephthalic acid (C_8_H_6_O_4_, Sigma-Aldrich), methanol (CH_3_OH, Merck), and N,N-dimethylformamide (DMF, HCON(CH_3_)_2_, Merck) were used for MIL-125 synthesis. Copper(II) nitrate trihydrate (Cu(NO_3_)_2_.3H_2_O, Merck) was used as Cu source for the synthesis of bimetallic MOF samples. Methylene blue (MB), a thiazine dye group, supplied by Merck was used as the synthetic dye for activity tests.

### 2.2. Catalyst preparation

The solvothermal method was followed for the preparation of MIL-125 (Ti). Terephthalic acid (3 mmol) and titanium isopropoxide (2 mmol) were added to dimethyl formamide (15 mL) and methanol (2 mL) solution. After stirring for 30 min, the mixture was subsequently moved into a stainless steel autoclave, where the reaction took place for 20 h at 150 °C. The crystals obtained were rinsed with ethanol and distilled water, and then dried at 80 °C for 2 h. The dry crystals were calcinated at 200 °C for 10 h for purification of the final sample from solvents.

The same technique was applied for the preparation of Cu-modified Ti-MOF. The concentration of metal center was calculated by weight percentage; 2 wt % of Cu(II) ions was added to titanium isopropoxide and the final amount was adjusted to 2 mmol.

### 2.3. Characterization

XRD patterns and crystallite sizes of nanoparticles were obtained with a Rigaku D/Max-2200 diffractometer (Cu Kα radiation with λ = 0.15418 nm). The measurements were carried out between 10° and 50° with 2°/min scanning speed.

FTIR analysis (Bruker Alpha) was performed by the KBr method to determine the structural properties of MOF crystals. Spectra were obtained between 400 and 4000 cm^−1^ wavenumber.

TGA (Hitachi STA 7200) was carried out under nitrogen atmosphere (200 mL/min) between 30 and 800 °C with a heating rate of 10 °C/min to determine the thermal behavior.

UV–vis DRS was carried out in the wavelength range of 200–800 nm using BaSO_4_ as reference by Shimadzu UV-2600 UV-VIS spectrophotometer.

SEM and EDX tests were conducted on fresh MOF crystals by scanning electron microscope-energy dispersive X-ray spectroscopy (Tescan Vega 3) at 10 kV and 10 k× magnification. The surface of the material was sputter coated with a gold/palladium layer prior to SEM and EDX measurements.

XPS analysis was performed using a Thermo Fisher Scientific K-alpha X-ray photoelectron spectrometer.

### 2.4. Photocatalytic activity measurements

Photocatalytic performance tests were carried out under visible light, utilizing a 105-W white fluorescent lamp emitting primarily at a wavelength of 530 nm. The reactions were conducted using 100 mL of 5 ppm aqueous MB solution within a reaction vessel having a diameter of 10 cm positioned beneath the lamp within a light chamber. Then 10 mg of photocatalyst was added to the MB solution in the reaction vessel followed by stirring for 1 h in the dark to attain equilibrium. Experiments without the presence of photocatalysts were conducted as well, revealing that there were no alterations in dye concentrations during irradiation. After 1 h, the zero-time reading was recorded and then the light was turned on and the degradation studies were performed under visible light for 300 min by magnetic stirring. A 5-mL sample of the reaction mixture was extracted at constant intervals and subsequently centrifuged to isolate the photocatalysts from the solution to determine dye concentration. Kinetic studies were performed by measuring the MB concentration at 0, 30, 60, 90, 120, 150, 200, 250, and 300 min. The effect of initial MB solution concentration on degradation efficiency was also studied at different MB concentrations varying in a range of 5–25 ppm. The effect of pH was also investigated by studying the MB degradation at different pH values in a range of 3–11. The degradation efficiencies were calculated by the following formula:


(1) $$\%\;\text{Degradation efficiency}=\frac{\left({\text{C}}_{0}-{\text{C}}_{\text{t}}\right)}{{\text{C}}_{0}}\times 100,$$

where C_0_ represents MB concentration at time 0 and C_t_ represents the concentration of MB at time t.

## 3. Results and discussion

### 3.1. Characterization results

**Figure 1:**
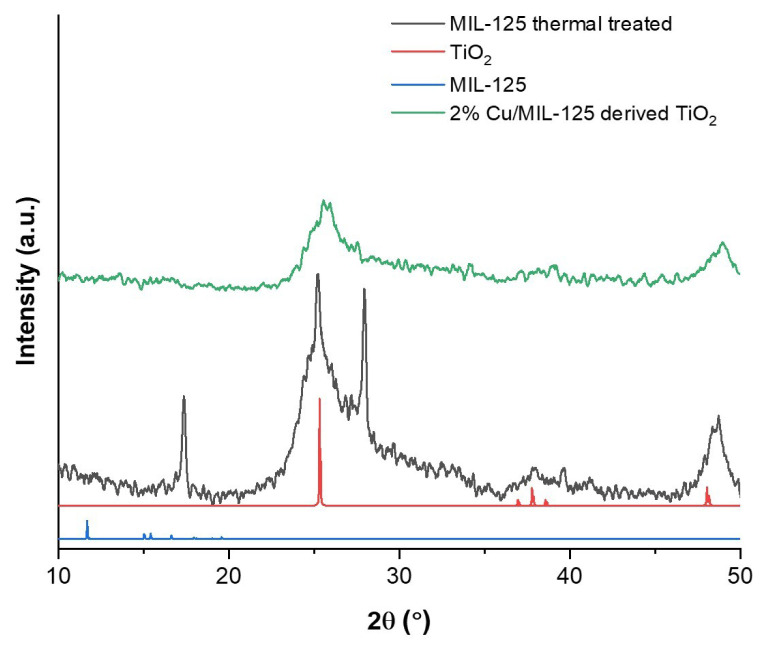
X-ray diffraction patterns of the photocatalysts.

XRD analyses were performed to provide insights into the nature of initial MIL-125 phases upon heat treatment and the change in TiO_2_ crystalline phase by the addition of Cu metal dopant. The XRD patterns of MIL-125 thermally treated and 2%Cu/MIL-125-derived TiO_2_ nanoparticles are presented in Figure 1. The anatase TiO_2_ XRD pattern was obtained from the Crystallography Open Database (Information Card No: 7206075) [27]. The XRD pattern of MIL-125 was obtained from the Cambridge Crystallographic Data Centre (deposition no. 751157) [28]. The software Vesta 3 was used for the calculation of XRD patterns from cif files of TiO_2_ and MIL-125 [29]. Figure 1 reveals that MIL-125 thermally treated comprises anatase TiO_2_ nanoparticles, having the same peaks at 25°, 37°, 38°, and 48°. The aim of heating at 200 °C under air atmosphere is to obtain TiO_2_ nanoparticles in different crystal structures without losing the original crystal structure of MIL-125. According to the XRD pattern, one of the specific peaks of MIL-125 at 16° does not vanish completely and rutile TiO_2_ peaks can be observed at the crystal at 28° [30]. The rutile and anatase phases of TiO_2_ nanoparticles exist in a mixture. The addition of 2% Cu to the MIL-125 structure weakened the crystal structure of MIL-125. Despite applying the same heat treatment procedure, the crystal structure is completely deformed and only anatase TiO_2_ peaks are clearly visible upon addition of 2% Cu, and the intensity of rutile phase decreases. Since the copper concentration is very low compared to titanium, copper oxide peaks are not observed.

The crystallite sizes of both samples were determined using the following Scherrer equation based on XRD patterns:


(2) $$D=\frac{k\lambda }{\beta cos\theta }$$

where *l* is the wavelength of the X-rays, *k* is a constant (1.5418), *b* is the peak width at half maximum, and *q* is the Bragg angle. The results indicate that incorporation of 2 wt % Cu into MIL-125 resulted in a decrease in the crystallite size of undoped TiO_2_ derived from MIL-125 from 7.0 nm to 5.5 nm. This shows that the doping TiO_2_ nanoparticles derived from MOF with Cu^2+^ ions ended up with slightly smaller nanosized titania crystallite particles. The diffraction pattern of the 2%Cu/MIL-125-derived TiO_2_ sample displays a broad profile, which is a characteristic of small crystallite sizes.

**Figure 2:**
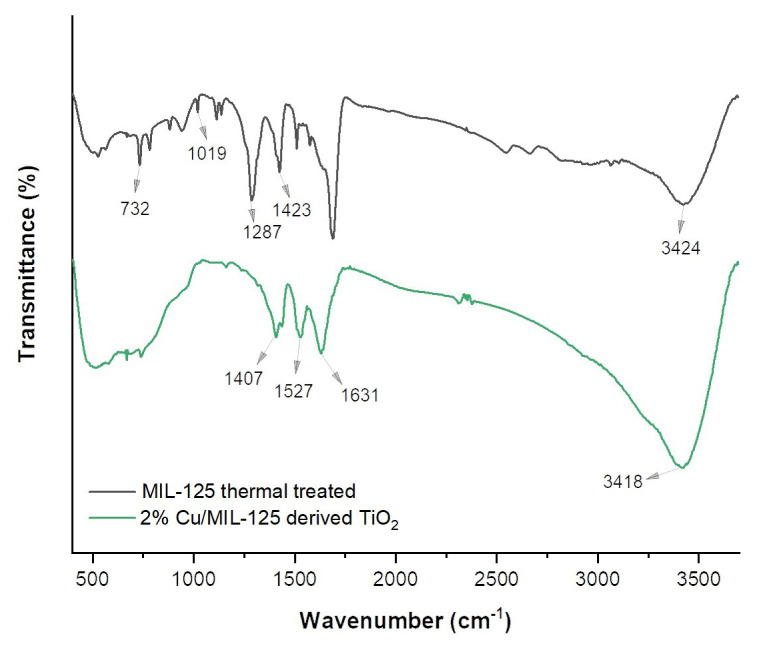
FTIR spectra of the photocatalysts.

Figure 2 presents the FTIR spectra of the photocatalysts. The broad band centered at 3400 cm^−1^ can be attributed to free solvent molecules trapped in the photocatalyst (O–H bond stretch). Strong bands at 1407–1423 cm^−1^ and 1509–1527 cm^−1^ correspond to the symmetric and asymmetric vibrations of the COO^−1^ groups. The strong bands at 1631 cm^−1^ can be attributed to the O–C–O vibrational stretching frequencies. This finding confirms the presence of the dicarboxylate linker in MIL-125 thermally treated and Cu-doped photocatalysts [31–33]. Absorption bands in the range of 1000–1600 cm^−1^ are the result of terephthalic acid stretching and bending [34]. The band at 1286–1291 cm^−1^ can be ascribed to the C–N groups, signifying the existence of coordinated DMF in the structure [35]. The bands at 1019 cm^−1^ and 732–738 cm^−1^ can be attributed to vibrations of the benzene rings. The bands between 450 and 800 cm^−1^ can be attributed to O–Ti–O vibrations [32]. This band gap could be damped in 2%Cu/MIL-125-derived TiO_2_ due to Cu^2+^ ions displaced by Ti^2+^ ions. This suggests that damping increases with increasing Cu^2+^ presence in the photocatalyst.

**Figure 3:**
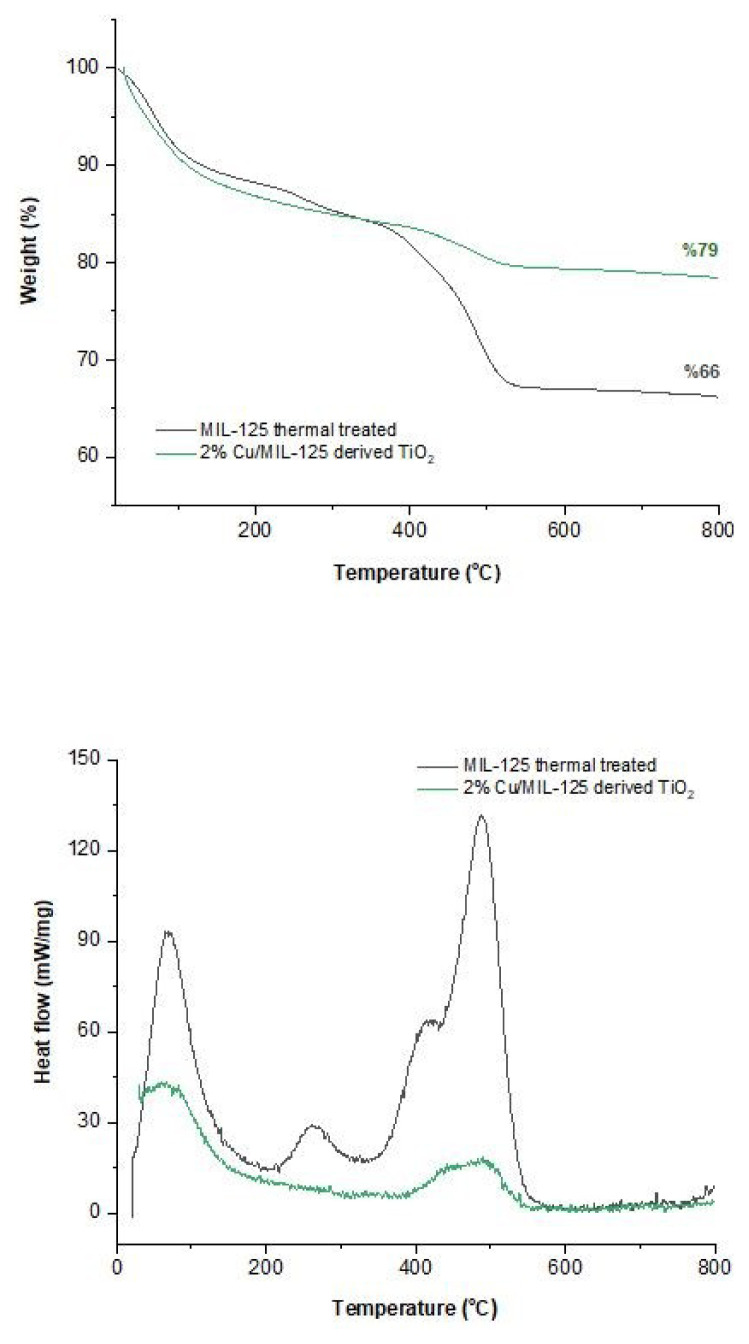
TGA and DTG profiles of the photocatalysts.

Figure 3 shows the TGA and DTG profiles of the heat-treated photocatalysts at 200 °C in stagnant air. The first weight loss up to about 100 °C observed for all samples is attributed to ethanol and water, which are used as washing solvents [36]. The weight loss of about 15% up to 250 °C can be ascribed to the removal of adsorbed water molecules from the surface of the MOF, the release of DMF from the MOF’s pores, and highly volatile components contained within the MOF [37]. The steep bend between 300 and 600 °C is probably due to terephthalic acid degradation [31]. While undoped MIL-125 thermally treated experienced a weight loss of 18% in this temperature interval, this rate decreased to 5% for the 2%Cu/MIL-125-derived TiO_2_ sample, indicating that integration of Cu^2+^ ions into the MIL-125 structure increased the thermal stability of the photocatalyst. The grafting of Cu^2+^ ions to the frameworks resulted in less stable crystals. After heat treatment, 2%Cu/MIL-125-derived TiO_2_ particles lose their MOF structure and turn into Cu/TiO_2_ nanoparticles. Total weight loss is 34% for bare MIL-125 thermally treated and 21% for 2%Cu/MIL-125-derived TiO_2_. The residuals of the materials are 66% and 79% for MIL-125 thermally treated and 2%Cu/MIL-125-derived TiO_2_ after 800 °C, respectively. According to these results, it can be suggested that incorporation of Cu into the MIL-125 thermally treated structure increases the thermal stability of TiO_2_ nanoparticles derived from MIL-125.

**Figure 4:**
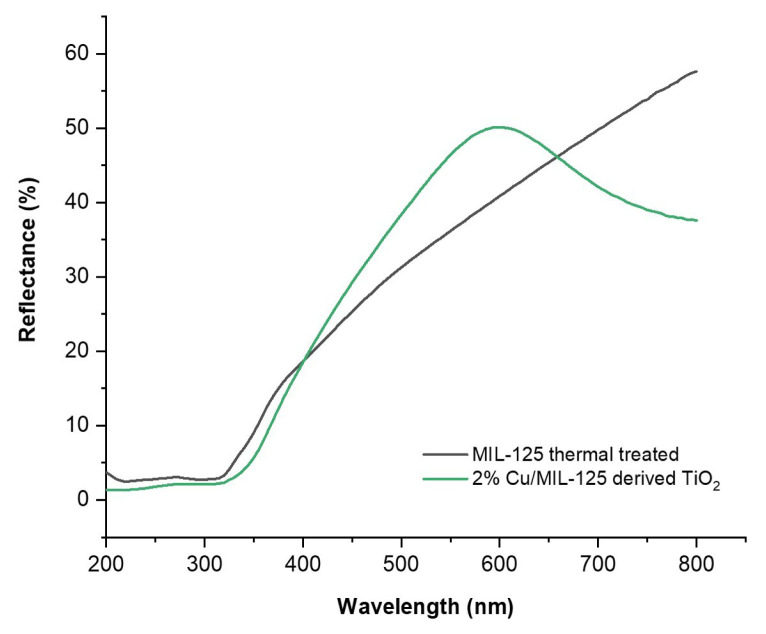
UV–vis reflectance spectra of the photocatalysts.

Figure 4 illustrates the UV–vis DRS profiles of both undoped and doped MIL-125 samples. A shift of the optical reflection edge into the visible light spectrum upon the addition of 2 wt % Cu is observed. In contrast to the undoped MIL-125 sample, which exhibits higher reflection within the visible range (>650 nm), the photocatalyst containing 2 wt % Cu^2+^ demonstrates lower reflection within this region, implying that addition of 2 wt % Cu into MIL-125 increased the photo response of the sample.

**Figure 5:**
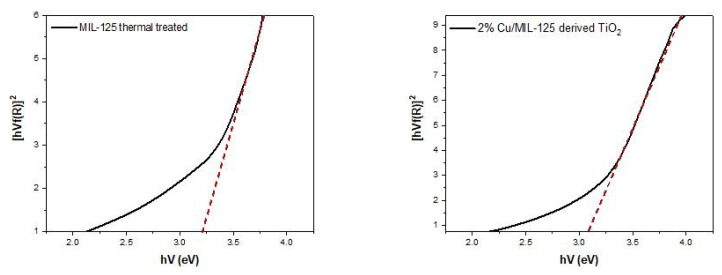
Tauc plots of bare MIL-125 thermally treated and 2%Cu/MIL-125-derived TiO_2_ for determination of band gap energy.

The direct band gap energy (E_g_) of MIL-125 samples was determined using the following equation:


(3) $${\left(\alpha \;\text{h}\upsilon \right)}^{2}=\text{A}(\text{h}\upsilon -E_{g})$$

where *hυ* is the photon energy, *α* is the absorption coefficient, *h* is Planck’s constant, E*_g_* is the direct band gap energy, and *A* is a proportional constant. The band gap values (*E_g_*) of each sample (Table 1) were determined by plotting a Tauc plot [38,39] and extending the linear segment of the curve to its x-intercept. Figure 5 represents the Tauc plot of both undoped and Cu-doped MIL-125. The band gap energy values are given in Table 1 as 3.21 and 3.07 for MIL-125 thermally treated and 2%Cu/MIL-125-derived TiO_2_, respectively. The results indicate that the band gap energy of the Cu-doped TiO_2_ nanoparticles derived from MIL-125 is significantly narrower than that of nondoped MIL-125. Using the equation l_g_ = 1240/E_g_, the optical absorption threshold wavelength (l_g_) of each sample was also calculated. The absorption threshold wavelength of the 2%Cu/MIL-125-derived TiO_2_ sample displayed a noticeable shift towards the visible light spectrum at 404 nm, in contrast to the undoped MIL-125, which exhibited an absorption threshold wavelength at 386 nm. It is clearly revealed that Cu^2+^-doped TiO_2_ nanoparticles derived from MIL-125 significantly enhance the absorption of visible light.

**Figure 6:**
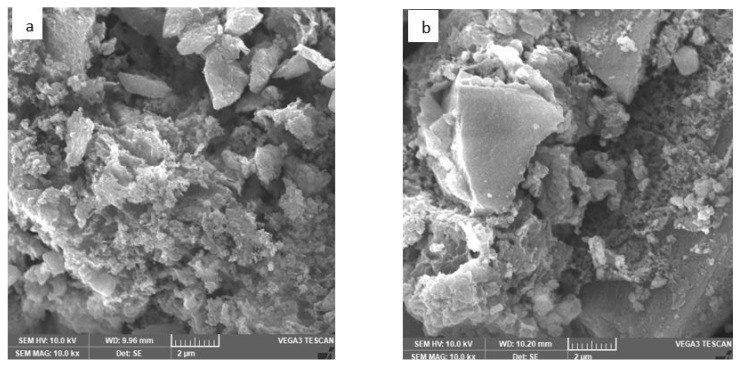
SEM images of a) MIL-125 thermally treated and b) 2%Cu/MIL-125-derived TiO_2_.

Figure 6 shows SEM images of the prepared MIL-125 thermally treated and 2%Cu/MIL-125-derived TiO_2_. As observed from the morphology examined at 2 μm by SEM, many random particles of different sizes are regularly scattered on the surface. In addition, the SEM images clearly depict that the photocatalysts are composed of block-like particles with well-defined crystalline structures, showcasing a variety of shapes [32,40].

**Figure 7:**
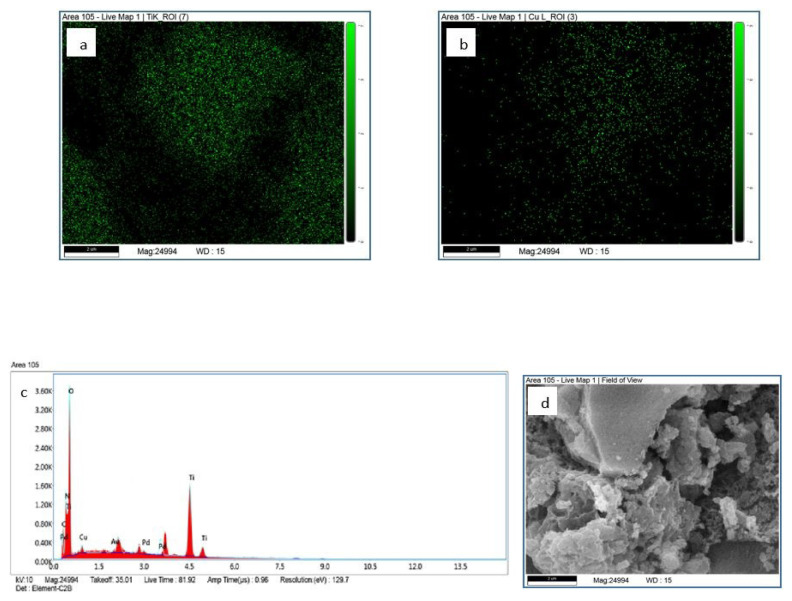
Elemental maps of a) Ti, b) Cu, c) EDX spectra, and d) field of view 2%Cu/MIL-125-derived TiO_2_.

EDX mapping was performed to provide additional verification of the scattering of Ti and Cu elements existing over 2%Cu/MIL-125-derived TiO_2_ photocatalyst. In Figure 7, the presence of Ti, Cu, O, N, and C elements in 2%Cu/MIL-125-derived TiO_2_ is observed, indicating that Cu is successfully bonded to the structure. Moreover, it is evident that there is more Ti than Cu in the structure of the photocatalyst.

**Figure 8:**
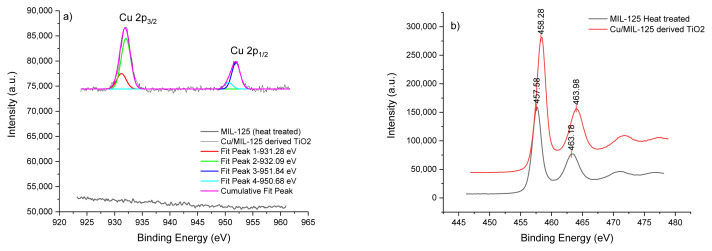
XPS spectra of MIL-125 heat treated and Cu/MIL-125-derived TiO_2_; Cu 2p peaks (a), Ti 2p peaks (b).

The XPS spectra are given in Figure 8. XPS data can provide information about the chemical composition of the nanoparticle surface. The XPS peaks of Cu are illustrated in Figure 8a. Binding energies of 932.08 and 952.08 eV were observed. These peaks correspond with Cu 2p_3/2_ and Cu 2p_1/2_, respectively. There are no Cu peaks in MIL-125 XPS spectra. Cu(I) peaks can be seen at 931.28 eV and Cu(II) peaks at 951.84 and 950.68 eV [41].

The XPS spectra of TiO_2_ nanoparticles can be observed in Figure 8b. The XPS peaks of anatase TiO_2_ nanoparticles can be observed at around 457 eV (2p_1/2_) and 463 eV (2p_3/2_) in the literature. The XPS spectra of the study are compatible with those in the literature [42].

### 3.2. Photocatalytic activity of the catalysts

**Figure 9:**
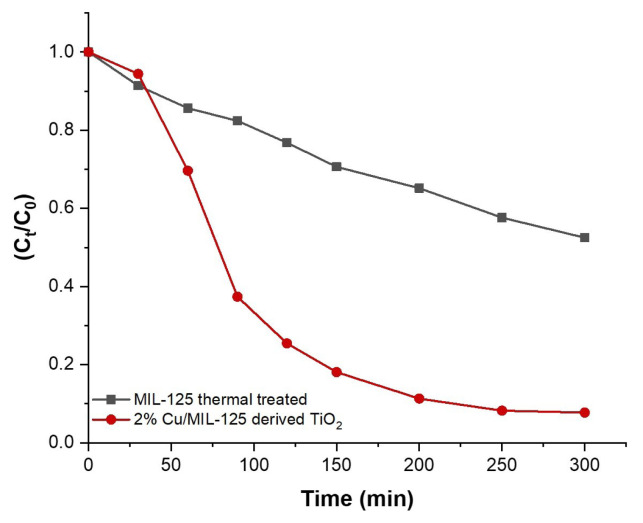
Photodegradation of MB as a function of irradiation time.

The present study investigated the photocatalytic degradation of MB, which is among the dyes frequently utilized in the textile industry. For this purpose, TiO_2_ nanoparticles derived from MIL-125 prepared by solvothermal method and doped with 2 wt % Cu^2+^ ions were tested under visible light in a batch-type photocatalytic reactor. In Figure 9, the photocatalytic degradation of MB under visible light exposure is presented for both undoped MIL-125 and 2%Cu/MIL-125-derived TiO_2_ samples, displaying changes over time. The degradation rates of the photocatalyst samples, calculated using Eq. (1), are also concisely listed in Table 1. The undoped TiO_2_ nanoparticles derived from MIL-125 showed 47.5% degradation at the end of 5 h, whereas the 2%Cu/MIL-125-derived TiO_2_ photocatalyst sample displayed a degradation efficiency of 92.3%, showing the strong correlation between the photocatalytic activity and the presence of Cu^2+^ ions in the nanostructure of TiO_2_. It is observed that more than 70% of the dye is degraded within 2 h of the photocatalytic process in the presence of doped MIL-125. It is clear that addition of Cu to the MOF framework drastically increased the photocatalytic degradation performance of TiO_2_ nanoparticles. The high activity profile of the Cu-doped photocatalyst may be attributed to the increased thermal stability, lower band gap energy, and smaller crystallite size of the sample.

Considering the crystallite sizes derived from the XRD analysis in the current investigation, Cu-doped MIL-125 with the lower crystallite size had a significantly higher dye degradation rate, whereas undoped MIL-125 with the larger crystallite size had a lower dye degradation performance. This result is in good agreement with several studies reporting that TiO_2_-based photocatalysts with lower crystallite sizes had significantly higher dye degradation efficiencies [43,44]. Abdelaal and Mohamed [43] reported that the photocatalytic degradation of MB dye exhibited an increasing sequence as follows: TiO_2_ < Pd/TiO_2_ < TiO_2_–CS < Pd/TiO_2_–CS and the crystallite size of each sample was in the order of TiO_2_ > Pd/TiO_2_ > TiO_2_–CS > Pd/TiO_2_–CS. They showed that catalysts with lower crystallite size presented higher photocatalytic activities. Xu et al. [44] studied the photocatalytic degradation reaction of reactive brilliant red under visible light using Ce, C-codoped titania nanoparticles. They also showed a noticeable correlation between crystallite size and activity, reporting that Ce, C-codoped titania had a crystallite size of 3.8 nm and photodegradation rate of 78.8%, whereas undoped titania had a crystallite size of 5.7 nm and photocatalytic activity of 19.5%.

Additionally, Cu-doped MIL-125 displayed considerably increased thermal stability compared to nondoped MIL-125. It is evident that the Cu-doped sample with considerably high thermal stability had 1.94 times greater activity performance than that of nondoped MIL-125. As suggested by Singh et al. [45], the high thermal stability of the Cu^2+^ metal ion integrated MIL-125 and its ability to withstand high temperatures make it an attractive and novel candidate as a photocatalyst as it will not degrade during photocatalysis under sunlight.

Furthermore, doping the MIL-125 structure with Cu^2+^ reduced the band gap of TiO_2_ nanoparticles derived from MIL-125. This reduction might have triggered the creation of a greater number of electron-hole pairs, thereby contributing to the enhancement of photocatalytic reactions. As depicted in the mechanism below [46], when photocatalyst particles absorb light matching their band gap energy, an electron undergoes excitation from the valence band to the conduction band, leading to the generation of electron-hole pairs. Subsequently, these segregated charges can engage with surface hydroxyl groups and adsorbed oxygen, triggering the generation of reactive oxygen species, which successively react with dyes, leading to complete breakdown of these compounds. For efficient photocatalytic reactions, it is crucial that the lifetimes of these electron-hole pairs are sufficiently prolonged to facilitate their migration to the photocatalyst’s surface. The incorporation of Cu^2+^ metal ions might have introduced additional trapping sites, affecting the duration of charge carrier lifetimes. When MIL-125 is doped with Cu^2+^ ion, photogenerated electrons are conveyed to the conduction band of Cu^2+^ sites, serving as new traps for these electrons, increasing the electron–hole pair lifetime. The transferred electrons participate in the surface redox reactions shown in Eqs. (6) and (7) [39,47]. This extension of the electron-hole pair lifetime might have increased the likelihood of interactions between the electron-hole pairs and reactive oxygen species.


(4) $$\text{Photocatalyst}+hn^{Ⓡ}{\text{e}}^{-}+{\text{h}}^{+}$$

(5) $${\text{Cu}}^{2+}+{\text{e}}^{-\;Ⓡ}\;{\text{Cu}}^{+}$$

(6) $${\text{O}}_{2}+{\text{e}}^{-\;Ⓡ}•{{\text{O}}_{2}}^{-}\;({\text{Cu}}^{+}+{{\text{O}}_{2}}^{Ⓡ}•{{\text{O}}_{2}}^{-}+{\text{Cu}}^{2+})$$

(7) $${\text{h}}^{+}+{\text{H}}_{2}{\text{O}}^{Ⓡ}•\text{OH}+{\text{H}}^{+}$$

(8) $$•{{\text{O}}_{2}}^{-}+{\text{H}}^{+Ⓡ}•{{\text{HO}}_{2}}^{Ⓡ}{\text{H}}_{2}{\text{O}}_{2}$$

(9) $${\text{H}}_{2}{\text{O}}_{2}+{\text{e}}^{-\;Ⓡ}2•\text{OH}$$

(10) $$\text{Dye}+•{\text{OH}}^{Ⓡ}{\text{intermediates}}^{Ⓡ}{\text{CO}}_{2}+{\text{H}}_{2}\text{O}$$

**Figure 10:**
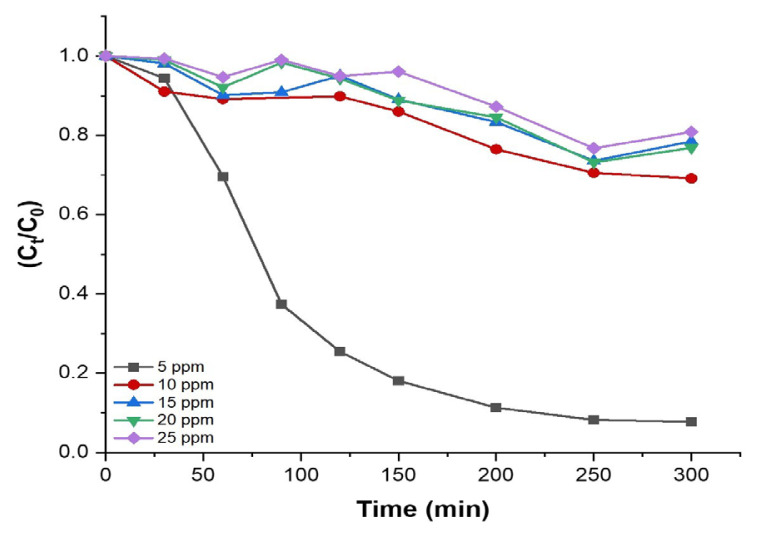
Photodegradation of MB by 2%Cu/MIL-125-derived TiO_2_ for different MB solution concentrations as a function of irradiation time.

As 2%Cu/MIL-125-derived TiO_2_ displayed the highest photocatalytic efficiency of greater than 90%, this sample was also tested for different MB solution concentrations (Figure 10). It is clearly observed that the degradation performance of the 2%Cu/MIL-125-derived TiO_2_ sample significantly decreases with an increase in initial dye concentration. The degradation rates of MB are 92%, 30%, 21%, 23%, and 19% for initial MB concentrations of 5, 10, 15, 20, and 25 ppm, respectively. The dye degradation rate declines with increasing initial dye concentration, primarily owing to the saturation of adsorption sites on the catalyst surface. At low dye concentration levels, a greater number of active sites become accessible on the surface, causing an abundance of hydroxyl radicals being generated. Conversely, at higher concentrations, degradation efficiency diminishes, caused by the creation of multiple layers of adsorbed dye molecules on the catalyst’s surface, preventing the reaction by obstructing the ability of dye molecules to access the catalyst’s surface [48]. In addition, the abundant MB molecules and their intermediates possibly obstruct the light absorption of TiO_2_ nanoparticles. The intermediates of MB might also compete for reactive radicals, leading to a reduction in the degradation efficiency of the photocatalyst, particularly at elevated MB concentrations [49]. Consequently, lower activity levels are obtained with increased initial concentrations of dye.

**Figure 11:**
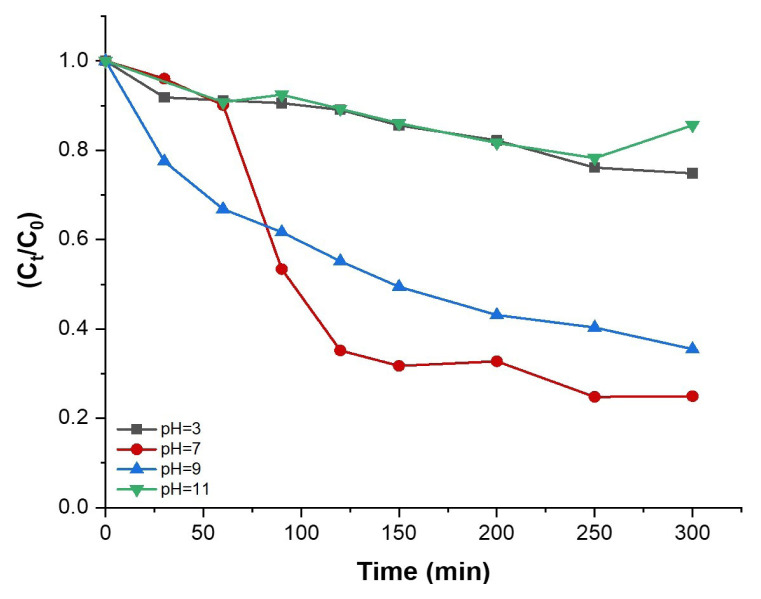
Photodegradation of MB by 2%Cu/MIL-125-derived TiO_2_ for different pH values as a function of irradiation time.

pH is a crucial parameter in shaping the adsorption characteristics of photocatalysts and determining the efficiency of dye degradation. TiO_2_ exhibits an amphoteric nature, allowing its surface to have a positive or a negative charge in response to changes in the solution pH [50,51]. Hence, alterations in pH can significantly influence the adsorption of dye molecules on the surface of TiO_2_, consequently leading to changes in the degradation rates [52,53]. In the current study, the influence of solution pH on dye degradation has also been explored within the pH range 3–11. As depicted in Figure 11, a clear trend emerges: as the pH is raised from 3 to 11, an initial rise in the degradation values is observed up to pH 7, which is then followed by a decline in the efficiency of degradation. The degradation rate of MB at the end of 5 h is 25.2, 75.1, 64.5, and 14.4 at pH values of 3, 7, 9, and 11, respectively. These results are similar to the findings obtained by Malini and Raj, who reported that the rose bengal dye degradation efficiency of CNS-TiO_2_ photocatalyst increased up to a pH value of 6; then a decrease was observed for higher pH values [52].

Heterogeneous photocatalytic dye degradation reactions are typically effectively described by the Langmuir–Hinshelwood rate model [39,47,54], which is expressed as


(11) $$r=-\frac{dC_{dye}}{dt}=\frac{k_{int}\times K\times C_{dye}}{1+K\times C_{dye}}$$

where *r* is the degradation rate, *C_dye_* is the dye concentration in solution, *k* is the reaction rate constant, *t* is the illumination time, and *K* is the adsorption coefficient of the reactant. When the initial concentration of the dye is very diluted, the term *K*′*C_dye_* becomes negligible and the Langmuir–Hinshelwood model can be rewritten to create the following pseudo-first-order kinetic equation:


(12) $$-ln\frac{C_{dye,t}}{C_{dy,0}}=k_{app}\times t$$

where *k_app_* is the apparent rate constant used in place of *k_int_* × *K*.

Table 2 summarizes apparent rate constants (*k_app_*) and coefficient of determination values (R^2^). The Cu^2+^ ion-doped sample demonstrated a 4.5 times higher rate constant than the undoped catalyst for MB degradation, confirming that the 2 wt % Cu^2+^ doping enhanced the photocatalytic activity. The literature comparison of the MB degradation kinetic parameters of different Cu/TiO_2_ photocatalysts under visible light is given in Table 3. It is evident that 2%Cu/MIL-125-derived TiO_2_ is a very efficient photocatalyst for MB degradation under visible light compared to the data in the literature.

## 4. Conclusions

In the current study, the aim was to design and develop Cu-modified TiO_2_ nanoparticles derived from MIL-125 prepared by solvothermal method. In order to boost the photocatalytic performance of TiO_2_ nanoparticles derived from MIL-125, 2 wt % Cu^2+^ ions were incorporated into the nodes of MOF frameworks to allow fine-tuning of the physicochemical properties of photocatalysts. The synthesized particles were tested by MB dye degradation reaction under visible light. Additionally, activity tests were carried out at five varying MB initial concentrations and four different pH values. According to the results obtained,

- Incorporation of 2 wt % Cu^2+^ ions into the MOF framework drastically increased the photocatalytic degradation performance of TiO_2_ nanoparticles.
- Increase in the initial dye concentration resulted in a decrease in degradation efficiency.
- Increasing the pH value in the range of 3–11 initially led to higher degradation rates until pH 7, after which the degradation rate began to decline.
- The high activity profile of Cu^2+^-doped photocatalyst might be attributed to the increased thermal stability, lower band gap energy, and smaller crystallite size of the sample.

## Figures and Tables

**Table 1:** Different properties of photocatalyst samples obtained from XRD and UV–vis DRS.

Photocatalysts	Band gap energy (eV)	Absorption edge wavelength (nm)	*Crystallite size, D (nm)
MIL-125 thermal treated	3.21	386	7.0
2%Cu/MIL-125 derived TiO_2_	3.07	404	5.5

* Determined by Scherrer equation from XRD.

**Table 2:** Reaction rate constants and degradation efficiencies.

Photocatalyst	MB degradation at the end of 5 h (%)	MB degradation kinetic parameters	
		k_app_ x 10^−3^ (min^−1^)	R^2^
MIL-125 thermal treated	47.5	2.2	0.9951
2%Cu/MIL-125 derived TiO_2_	92.3	9.8	0.9504

**Table 3:** The literature comparison of MB degradation kinetic parameters of different Cu/TiO_2_ photocatalysts under visible light.

Photocatalyts	k_app_ x 10^−3^ (min^−1^)	Reference
10Cu^2+^-TiO_2_/FTO	3.0	[47]
Cu/TiO_2_	5.3	[39]
PcTcCu–TiO_2_/O_2(g)_	1.7	[55]
Cu-TiO_2_	4.0	[56]
Copper-doped powder	1.4	[57]
2%Cu/MIL-125 derived TiO_2_	9.8	This study
